# Effects of non-pharmacological interventions on body composition and physical function in older women with sarcopenic obesity: a meta-analysis

**DOI:** 10.3389/fpubh.2025.1718720

**Published:** 2025-12-12

**Authors:** Chuang Zeng, Hengxu Du, Junqiu Zheng, Yunting Wang, Kechen Liu, Zhilin Chen

**Affiliations:** 1 School of Nursing, University of South China, Hengyang, Hunan, China; 2 Heyou International Health System, Foshan, China; 3 The Affiliated Panyu Central Hospital of Guangzhou Medical University, Guangzhou, China; 4 Bao'an Center Hospital of Shenzhen, Shenzhen, China

**Keywords:** sarcopenic obesity, non-pharmacological treatment, aged, exercise therapy, meta-analysis

## Abstract

**Background:**

Sarcopenic obesity is characterized by excessive fat mass accompanied by sarcopenia, resulting in combined health risks associated with both conditions. Currently, no standardized or effective treatment approach has been established. The health risks and potential for physical disability associated with sarcopenia increase with age, and older women face greater challenges than men due to the accelerated muscle loss following menopause.

**Objective:**

Given the absence of standardized or effective treatments, this systematic review aimed to evaluate which non-pharmacological interventions can effectively improve body composition and physical function in older women (aged ≥60 years) with sarcopenic obesity.

**Design:**

Systematic review and meta-analysis.

**Setting and participants:**

Older women aged 60 years and above.

**Methods:**

A comprehensive search of four electronic databases (PubMed, Web of Science, Embase, and the Cochrane Library) was conducted up to April 2025. A total of 11 studies were included in the meta-analysis, comprising 10 randomized controlled trials (RCTs) and one quasi-experimental study. Although the latter did not employ random allocation, it was included due to the rigor of its intervention design, the completeness of its data, and its relevance to the research question. The primary outcomes measured included key indicators of body composition and physical function. The methodological quality of the included studies was assessed using the Cochrane Risk of Bias 2.0 (RoB 2) tool. To determine the certainty of evidence for the main outcomes, the GRADE approach was applied, evaluating five domains: risk of bias, inconsistency, indirectness, imprecision, and publication bias.

**Results:**

A total of 11 studies (10 randomized controlled trials and 1 non-randomized controlled trial) involving 532 participants were included. Meta-analysis revealed that exercise and other non-pharmacological interventions significantly reduced body fat percentage (WMD = −1.85, 95% CI: −3.35 to −0.36, *p* < 0.05) and significantly improved appendicular free fat mass (WMD = 0.64, 95% CI: 0.60–0.68, *p* < 0.001), skeletal muscle index (WMD = 0.64, 95% CI: 0.43–0.86, *p* < 0.001), and handgrip strength (WMD = 2.96, 95% CI: 1.76–4.16, *p* < 0.001). No significant differences were observed in gait speed, waist circumference, or body mass index (all *p* > 0.05). Subgroup analyses suggested that combined interventions were more effective than single interventions in reducing body fat, and short-term programs were superior to long-term ones. For muscle mass outcomes, single resistance training showed more consistent benefits in improving Appendicular Free Fat Mass, whereas the addition of nutritional supplementation conferred no significant extra advantage.

**Conclusion:**

Future studies on sarcopenic obesity should adopt the latest international consensus criteria (e.g., European Working Group on Sarcopenia in Older People 2, Asian Working Group for Sarcopenia 2019) to enhance the comparability and clinical applicability of findings. Current evidence indicates that exercise interventions, particularly resistance training, serve as the cornerstone for improving handgrip strength and functional outcomes such as gait speed. Various non-pharmacological approaches have also been validated in improving body composition indicators (Body Fat Percentage, Appendicular Free Fat Mass, and Skeletal Muscle Index). The synergistic effects of combined exercise and nutritional interventions on muscle mass and strength warrant further investigation, especially to determine the optimal modality and dosage in women with sarcopenic obesity. Future intervention strategies should be systematically designed to integrate short-term intensive programs with long-term maintenance, thereby maximizing therapeutic benefits and improving clinical feasibility.

## 1 Introduction

Obesity is a major global public health concern affecting individuals across all age groups, with its prevalence steadily increasing worldwide. This trend is particularly evident among older adults (1). Obesity is associated with numerous chronic diseases, including cardiovascular disease, diabetes, and certain types of cancer, and contributes to reduced quality of life and premature mortality (2). At the same time, sarcopenia, an aging-related syndrome, is also highly prevalent in older populations. First proposed by Rosenberg in 1989, sarcopenia was formally defined in 2010 by the European Working Group on Sarcopenia in Older People (EWGSOP) as the progressive loss of skeletal muscle mass and strength with age (3). Both sarcopenia and obesity are independently associated with physical disability in older adults. With aging, however, body composition undergoes critical changes, characterized by increased total and visceral fat mass alongside progressive declines in muscle mass and strength. When excessive fat accumulation coexists with sarcopenia, this combined condition is termed sarcopenic obesity (SO) (4).

The 2022 European Society for Clinical Nutrition and Metabolism and European Association for the Study of Obesity consensus statement emphasized that SO is distinct from either sarcopenia or obesity alone. This condition not only encompasses the individual health risks of both but may also result to more severe adverse outcomes (5). Epidemiological evidence suggests that older women face approximately three times the risk of SO compared with older men (6). In a cohort study of older women, Rolland et al. (7) reported that the odds ratios (ORs) for functional impairment (e.g., stair climbing) were 1.47 for women with sarcopenia, 1.79 for women with obesity, and as high as 3.60 for women with SO, highlighting the substantially increased risk of disability, particularly in females. This pronounced sex difference may be linked to the natural age-related decline in muscle mass, strength, and physical function.

From a clinical perspective, older women are recognized as an important target population for SO prevention and management. Compared with men, they generally present with lower baseline muscle mass and strength (8), and demonstrate a faster conversion of lean mass to fat mass with weight gain (9). Postmenopausal hormonal changes further exacerbate these risks: a sharp decline in estrogen triggers increased hypothalamic secretion of gonadotropin-releasing hormone (GnRH), which in turn elevates pituitary follicle-stimulating hormone (FSH) secretion (10). FSH binds to receptors expressed in visceral adipose tissue, activating the Gαi inhibitory signaling pathway and promoting lipogenesis (11). This endocrine–metabolic axis may accelerate fat accumulation and aggravate muscle loss in older women, thereby contributing to their higher prevalence of SO. Moreover, women tend to engage in lower levels of physical activity compared with men, further increasing their risk (12). Given that women constitute a larger proportion of the older adult population and have a longer life expectancy, they face greater burdens of functional limitations and disability during extended life spans (13).

SO has drawn increasing attention in recent years and is often described as an “obesity paradox,” since the coexistence of muscle loss and obesity challenges traditional perspectives (14). This dual burden makes treatment particularly complex, as patients must simultaneously gain muscle and lose fat. Although research into treatment strategies is growing, exercise combined with nutritional interventions remains the most widely studied non-pharmacological approach. However, standardized treatment guidelines or specific pharmacological therapies for SO are still lacking (15).

Resistance training (RT) is regarded as a cornerstone of exercise interventions for older adults. It offers structured, progressive, and relatively high-intensity training that can induce rapid adaptations. Numerous recent studies have demonstrated its efficacy in improving muscle strength, muscle mass, and overall physical function in older individuals (16). Accordingly, RT has been widely implemented in the management of sarcopenia and obesity and is considered a foundational non-pharmacological intervention. However, given the complexity of SO as a combined condition, it remains uncertain whether RT alone provides definitive therapeutic benefits. Evidence suggests that combined interventions incorporating both aerobic and resistance training may be more effective in improving body composition, promoting muscle gain while reducing fat mass (17). Such multimodal programs can simultaneously target cardiovascular fitness and muscle strengthening, yielding broader metabolic and functional improvements. Additionally, low-load RT combined with blood flow restriction or resistance band training has been explored, showing potential benefits in enhancing muscle mass and reducing fat while minimizing injury risk (18). Beyond exercise, other non-exercise modalities, such as nutritional supplementation and electrical stimulation have been investigated, either to augment exercise outcomes or to provide alternatives for those with limited mobility.

Despite growing interest, systematic reviews of SO interventions remain limited. Some studies suggest that RT effectively improves total fat-free mass (TFFM), yet its effects on body fat percentage (BF%) and body mass index (BMI) remain inconsistent (19). Such variability underscores the uncertainty surrounding the efficacy of RT in SO management and highlights the need for comprehensive evidence synthesis. In addition, the long-term effectiveness of such interventions may depend not only on physiological adaptations but also on psychological mechanisms—such as mindset, sense of agency, environmental support, and self-regulatory capacity—that influence adherence and behavior maintenance. From an educational standpoint, this raises an important reflection on how intervention programs can foster self-management competence and encourage individuals to sustain health-promoting behaviors beyond structured participation. Although the present review focuses primarily on physiological and functional outcomes, acknowledging these perspectives helps to contextualize intervention sustainability and identify directions for future research. To date, no systematic review has comprehensively evaluated all available non-pharmacological interventions for SO specifically in older women. Therefore, an updated systematic review and meta-analysis is warranted to assess the comparative efficacy of these interventions. The present study aims to synthesize recent randomized controlled trials (RCTs) to evaluate the effectiveness of non-pharmacological interventions for managing SO in older women.

## 2 Methods

### 2.1 Registration

This systematic review and meta-analysis was conducted in accordance with the Preferred Reporting Items for Systematic Reviews and Meta-Analyses (PRISMA) guidelines (20). The study protocol was prospectively registered on the PROSPERO platform (registration number: CRD420251000692).

### 2.2 Search strategy

Two independent reviewers (CZ and HXD) systematically searched four electronic databases: PubMed, Web of Science, EMBASE, and the Cochrane Library, from their inception to April 2025. The search strategy combined Medical Subject Headings (MeSH) and free-text terms using Boolean operators. The following terms were applied: “sarcopenia” OR “sarcopenic” AND (“obesity” OR “obese” OR “overweight”) AND (“female” OR “women”) AND (“exercise intervention” OR “nutritional intervention” OR “electrical stimulation therapy”). The complete search strategies for each database are provided in Appendix Table 1.

### 2.3 Eligibility criteria

The inclusion and exclusion criteria were established according to the PICOS framework: participants, interventions, comparisons, outcomes, and study design.

*Participants (P)*: Older women (≥60 years) diagnosed with SO. Studies were excluded if participants had severe comorbidities such as cancer, multiple sclerosis, stroke, or cognitive impairment.

*Interventions (I)*: Non-pharmacological interventions, including resistance training, weight training and/or elastic band training, nutritional interventions, and electrical stimulation therapies.

*Comparisons (C)*: Control groups receiving usual care or health education.

*Outcomes (O)*: Studies that reported outcomes related to body composition and/or physical function. Studies with unmatched or irrelevant outcome measures were excluded.

*Study design (S)*: Randomized controlled trials (RCTs) and quasi-experimental studies were included. Case reports, reviews, and systematic reviews were excluded.

Additional criteria: Only peer-reviewed articles published in English before April 15, 2025, were included. Reviews, pathological reports, conference abstracts, and letters were excluded.

### 2.4 Data extraction

All retrieved records were managed using EndNote X9 software to remove duplicates. Two independent reviewers (CZ and HXD) initially screened the titles and abstracts. Full-text screening was subsequently performed by one reviewer, with disagreements resolved by consultation with a third independent adjudicator. Extracted data included study characteristics (first author, year of publication, country), participant characteristics [sample size, age, body mass index (BMI)], definitions of SO, body composition assessment tools, details of the intervention (type, comparator, frequency, intensity, and duration), and reported outcome measures. A summary of the extracted data is provided in Table 1.

**Table 1:** Summary of diagnostic frameworks and diagnostic criteria for SO used across the included studies.

Study	Region	Diagnostic framework	Diagnostic criteria
Aubertin et al. (22)	Canada	Researcher-defined	AFFM/height^2^ < 6.87 kg/m^2^ + BF > 40% (DXA)
Gadelha et al. (19)	Brazil	Researcher-defined	SO index = −13.012 + 16.737 × height (m) + 0.07231 × FM (kg)
Kim et al. (33)	Japan	AWGS 2014 (Japan-specific)	BF ≥ 32% (DXA) + (SMI < 5.67 kg/m^2^ OR HGS < 17 kg OR GS < 1.0 m/s)
Wittmann et al. (32)	Germany	EWGSOP 2010	ASM/height^2^ below cut-off + high FM (DXA)
Vasconcelos et al. (25)	Brazil	Researcher-defined	BMI ≥ 30 kg/m^2^ + HGS ≤ 21 kg
Huang et al. (26)	Taiwan	Researcher-defined	SMI < 27.6% (BIA) + BF > 30%
Liao et al. (27)	Taiwan	EWGSOP 2010	SMI < 7.15 kg/m^2^ + BF > 30%
Jinkee et al. (2017)	Korea	Researcher-defined (Korean)	ASM/weight < 25.1% + BMI ≥ 25 kg/m^2^
Liao et al. (29)	Taiwan	Researcher-defined	SMI < 27.6% + BF > 30%
Nabuco et al. (30)	Brazil	Researcher-defined	ALST < 15.02 kg + BF ≥ 35%

### 2.5 Risk of Bias assessment

**Figure 1:**
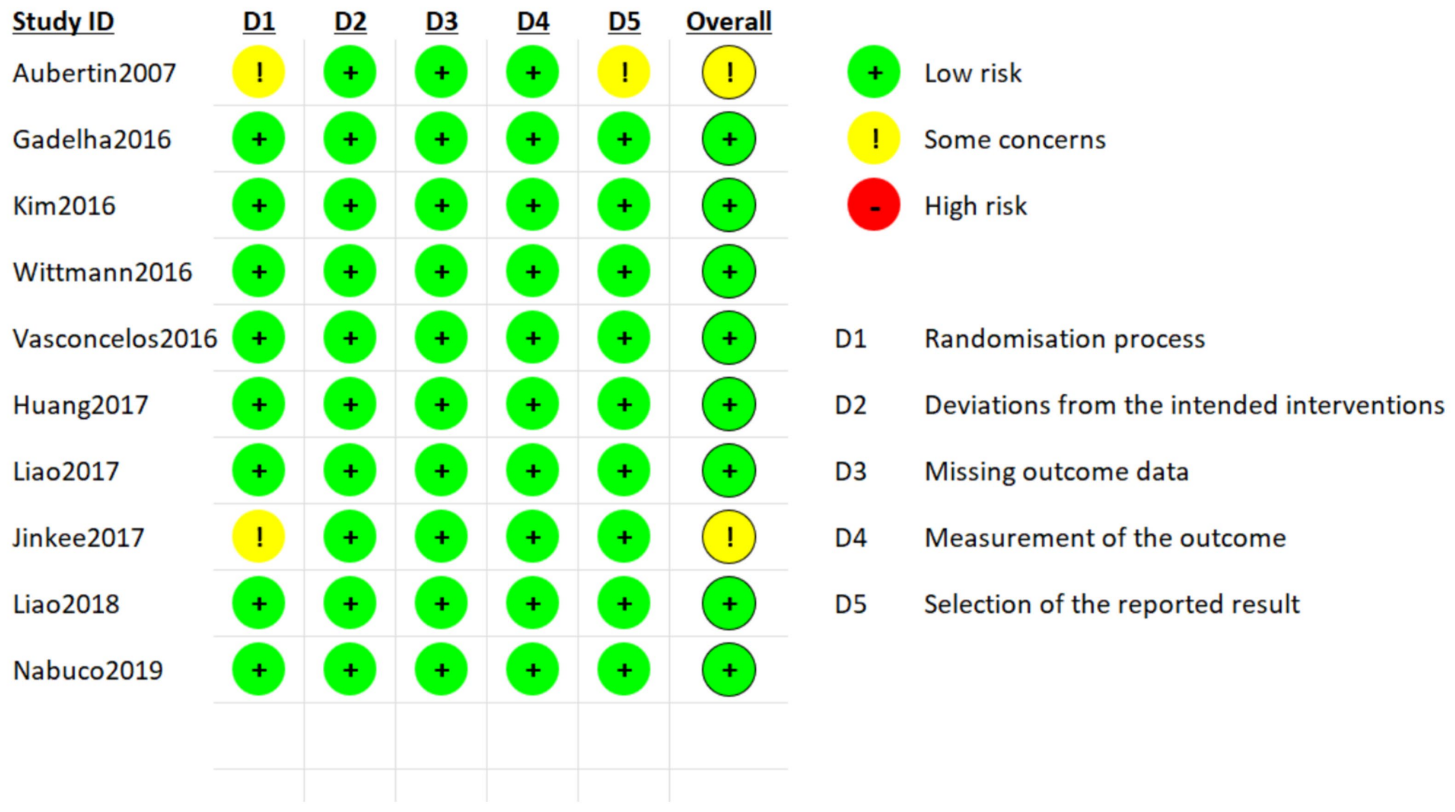
Risk of bias summary across five domains for all included studies.

**Figure 2:**
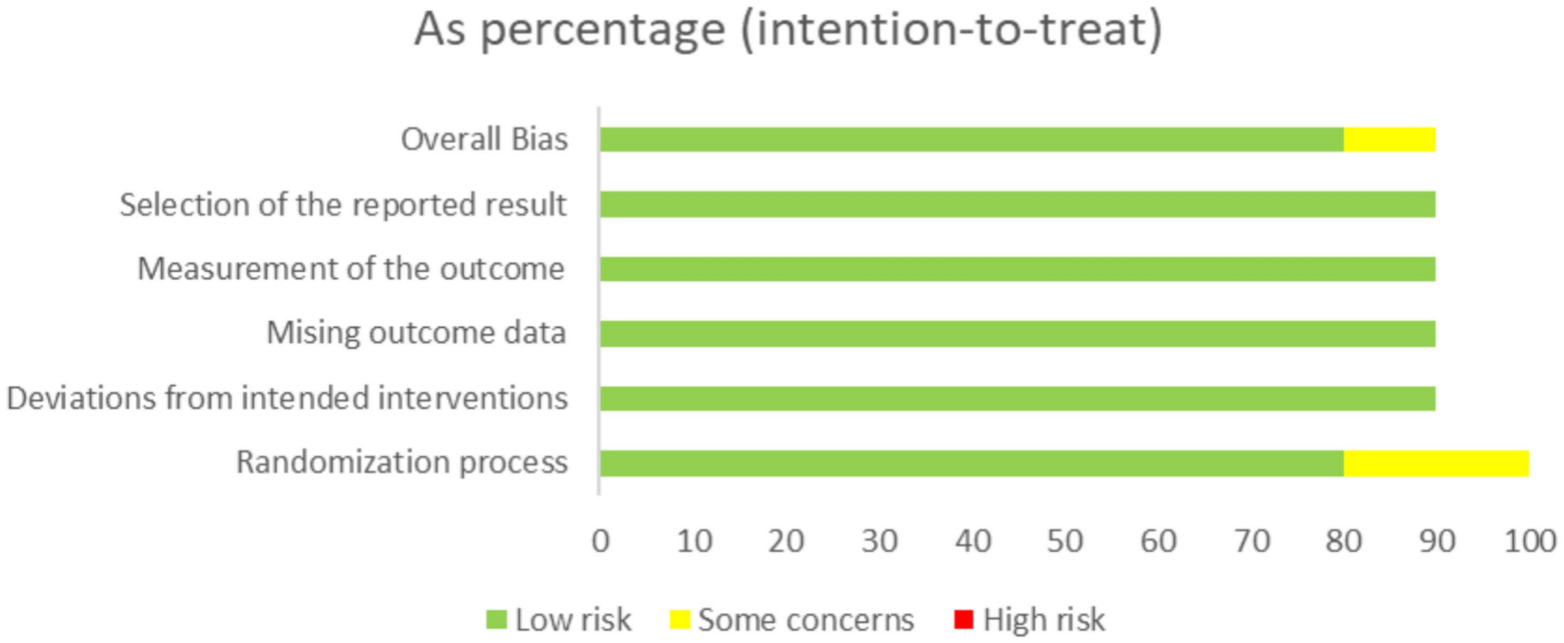
Percentage of risk of bias assessments.

The methodological quality of the included studies was assessed using the Cochrane Risk of Bias tool 2 (RoB 2) (21) (Figure 1). Two reviewers independently evaluated the risk of bias across five domains. Each domain was rated as “low risk,” “high risk,” or “some concerns” at the study level. Any disagreements were resolved through discussion, with explicit reference to evidence from the original studies. When consensus could not be reached, a third independent reviewer was consulted to provide adjudication (Figure 2).

### 2.6 Evidence grading

The certainty of the evidence was evaluated using the GRADE (Grading of Recommendations Assessment, Development and Evaluation) approach (see Appendix PDF for details).

### 2.7 Data analysis

All statistical analyses were performed in RStudio (version 4.3.1) using the “meta” and “metafor” packages. For continuous outcomes, effect sizes were expressed as standardized mean difference (SMD) or mean difference (MD) with 95% confidence intervals (CIs). Statistical heterogeneity across studies was assessed using Cochran’s Q test and the *I*^2^ statistic. Significant heterogeneity was defined as *I*^2^ > 50% and *p* < 0.1, in which case a random-effects model was applied; otherwise, a fixed-effects model was used.

To assess the robustness of the findings, sensitivity analyses were conducted by sequentially excluding individual studies. Where appropriate, descriptive analyses were performed to supplement the quantitative results. Subgroup analyses were performed based on intervention type and outcome category. Publication bias was evaluated using Egger’s regression test and visual inspection of funnel plots.

## 3 Results

### 3.1 Study selection

**Figure 3:**
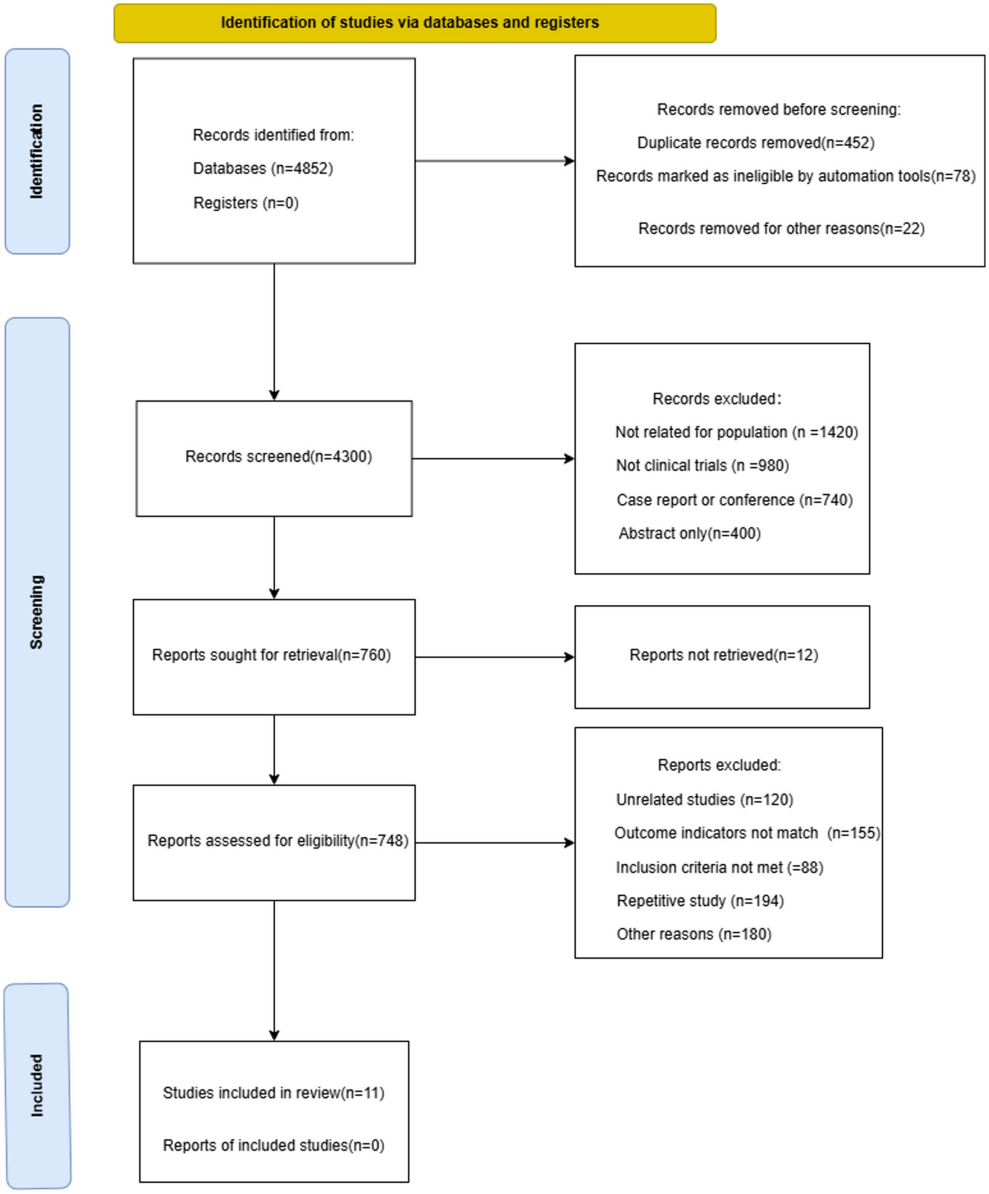
PRISMA flow diagram of the study selection process.

According to the predefined search strategy, a total of 4,852 records were initially retrieved from the four databases. After removal of 452 duplicates, 4,300 studies were screened by title and abstract. Subsequently, 748 full-text articles were assessed for eligibility. Finally, 11 studies (19, 22–32) were included for analysis (28, 31). The detailed study selection process is illustrated in Figure 3.

### 3.2 Characteristics of included studies

This meta-analysis included 10 randomized controlled trials (RCTs) and 1 quasi-experimental study, with a total of 516 participants (265 in intervention groups and 251 in control groups). The included studies were published between 2007 and 2023 and were conducted in Canada, Japan, Germany, Brazil, Taiwan, China and South Korea.

Most interventions were based on resistance training, either alone or in combination with other modalities. Five studies employed resistance training alone [e.g., (19, 25–27, 29)]. Three studies implemented combined interventions, such as exercise plus nutrition (33), aerobic plus resistance training (Jinkee et al., 2017), and protein supplementation plus resistance training (30). One study investigated electrical muscle stimulation (EMS) (32), another examined isoflavone supplementation (22), and one quasi-experimental study (Shumeng et al., 2023) evaluated aquatic exercise, which differed substantially from land-based resistance, aerobic, or nutritional interventions in terms of training environment, muscle activation patterns, and joint loading. Its inclusion adds representativeness and diversity to the present analysis.

The duration of interventions ranged from 10 to 24 weeks, with most studies (*n* = 9) lasting 12 weeks. Training frequency was generally two to three sessions per week, although some studies adopted mixed-frequency designs (e.g., Jinkee et al., 2017: aerobic five sessions per week plus resistance training three sessions per week). All included studies clearly described their intervention protocols and implemented supervision mechanisms to ensure adherence.

Outcome measures encompassed body composition (e.g., BF%, BMI, SMI) and functional performance (e.g., gait speed), providing sufficient data for subsequent meta-analysis Table 2 summarizes general study information and key characteristics. The detailed characteristics of the included studies are presented in Table 2, which is provided at the end of the manuscript.

**Table 2:** Characteristics of included studies.

First author,year	Region	sample(I/C)	duration time	Frequency	Intensity	Study design	Intervention	Control	Outcome Measure	Included in Main Analysis (Yes/No)
Aubertin et al. (22)	Canada	12//6	24 weeks	4 capsules /day	Not applicable	RCT	Isoflavone supplementation	Placebo(non-intervention)	3, 6, 8	Yes
Gadelha et al. (19)	Brazil	69/64	24 weeks	3 sessions /week	RPE 10–13	RCT	Resistance training	Placebo(non-intervention)	2, 3, 4, 5, 8	Yes
Kim et al. (33)	Japan	36/34	12 weeks	2 sessions /week	Moderate	RCT	Exercise + nutrition	health education every 2 weeks	3, 5, 6, 7, 8, 15	Yes
Wittmann et al. (32)	Germany	25/25	24 weeks	1 session /week	Moderate–High (WB-EMS)	RCT	Whole-body electromyostimulation (WB-EMS&P)	Placebo(non-intervention)	2, 7, 15, 20	Yes
Vasconcelos et al. (25)	Brazil	14/14	10 weeks	2 sessions /week	Moderate–high (Borg); 1-RM based; add speed wk5+	RCT	Resistance training	Monitoring health conditions via telephone	5, 7, 21, 22, 23	Yes
Huang et al. (26)	Taiwan	18/17	12 weeks	3 sessions /week	Moderate(RPE13); yellow band start; progressed by color	RCT	elastic band resistance training	a 40-min lesson about the exercise concept	1, 3, 24, 25, 26, 27	Yes
Liao et al. (29)	Taiwan	25/21	12 weeks	3 sessions /week	Moderate(RPE13); Theraband: yellow→silver (1.3–6.0 kg)	RCT	Elastic resistance training	Placebo(non-intervention)	1, 2, 3, 7, 8, 15, 16, 17, 18, 28, 29, 30	Yes
Jinkee et al. (2017)	Korea	25/25	24 weeks	Aerobic: 5 sessions /week; Resistance: 3 sessions /week	Aerobic: RPE 13–15 → ≥15; RT: 2–3 × (8–11 → 12–15 reps), 1-min rest	RCT	resistance and aerobic exercise	Placebo(non-intervention)	3, 15, 17, 31, 32, 33, 34	Yes
Liao et al. (29)	Taiwan	33/23	12 weeks	3 sessions /week	Moderate (RPE 13); yellow band upward progression	RCT	Resistance training	Placebo(non-intervention)	6, 7, 12, 16, 17, 35, 36	Yes
Nabuco et al. (30)	Brazil	13/13	12 weeks	3 sessions /week	Load increased 2–5% (upper) or 5–10% (lower); adjusted weekly when 3 sets × 12 reps achieved in 2 sessions.	RCT	Resistance training plus protein	Placebo(non-intervention)	3, 9, 12, 13	Yes
Shumeng et al. (2023)	Japan	9//11	12 weeks	3 sessions /week	RPE 12–16 (moderate to high intensity).	Non-RCT(excluded from primary analysis)	Aquatic exercise program	Placebo(non-intervention)	3, 6, 7, 19, 37, 38	No

1: Total Fat Mass (kg), 2: Total Muscle Mass (kg), 3: Body Fat Percentage (%), 4: BMI (kg/m^2^), 5: Waist Circumference (cm), 6: Skeletal 7: Muscle Index (kg/m^2^), 8: Gait Speed (m/s), 9: Appendicular FFM / ASM (kg), 10: Arm Muscle Index (kg/m^2^), 11: Leg Muscle Index (kg/m^2^), 12: Appendicular Lean Mass (kg), 13: Upper Limb Muscle Quality (ratio), 14: Lower Limb Muscle Quality (ratio), 15: Lean Soft Tissue (LST, kg), 16: Handgrip Strength (kg), 17: Lower Limb Strength (kg), 18: Chair Stand Test (reps/30s), 19: Flexibility (cm), 20:2-Minute Step Test (reps), 21: Peak Torque (Nm), 22: SPPB Score (0–12), 23: SF-36 Physical Function (score), 24: Muscle Power (W), 25: Total BMD (g/cm^2^), 26: BMD T-score, 27: BMD Z-score, 28: Bone Mineral Density (unspecified type), 29: Single-leg Balance (s), 30: TUG (Timed Up and Go) (s), 31: Max Gait Speed (m/s), 32: Left Grip Strength (kg), 33: Chair Stand Up, 34: Combined Strength (kg), 35: MVC (kg or Nm), 36: Lower limbs FFM (kg), 37: Lower limbs Fat Mass (kg), 38: Lower limbs Fat % (%).

### 3.3 Meta-analysis

#### 3.3.1 Analysis of body fat percentage

**Figure 4:**
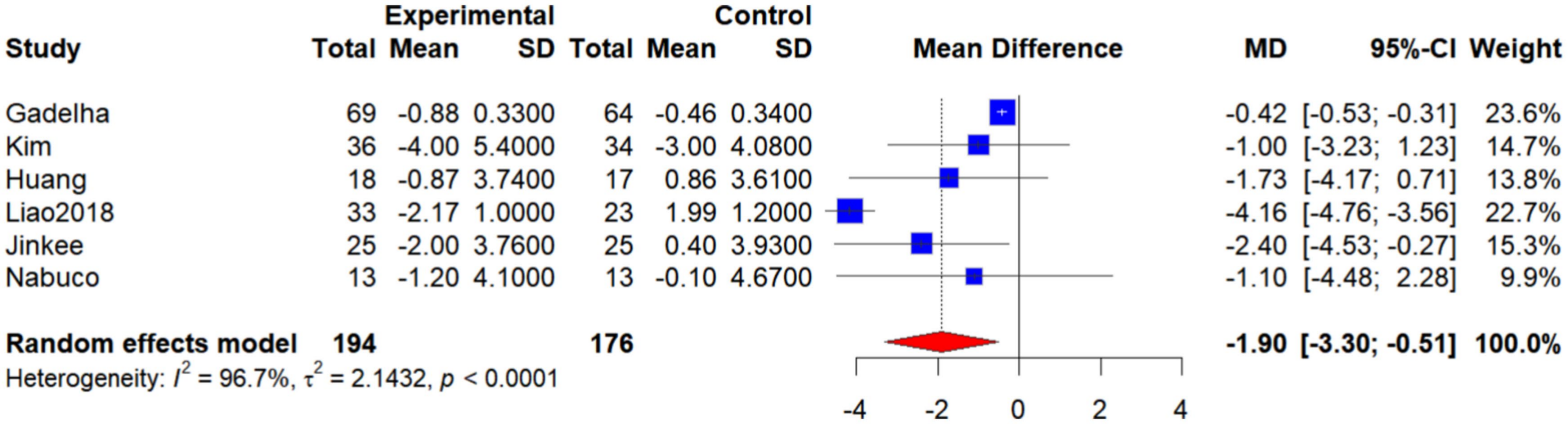
Forest plot of the effect of interventions on BF%.

Six studies (19, 26, 29, 30, 33) (Jinkee et al., 2017) evaluated the effects of exercise interventions on body fat percentage (BF%) in older women. Due to substantial heterogeneity among effect sizes (*I*^2^ = 96.7%, *p* < 0.0001), a random-effects model was employed. The pooled analysis revealed that BF% was significantly lower in the intervention group compared with the control group (WMD = −1.90, 95% CI: −3.30 to −0.51, *p* < 0.05), indicating that exercise interventions exert a significant beneficial effect on reducing BF% in older women with SO (Figure 4).

**Figure 5:**
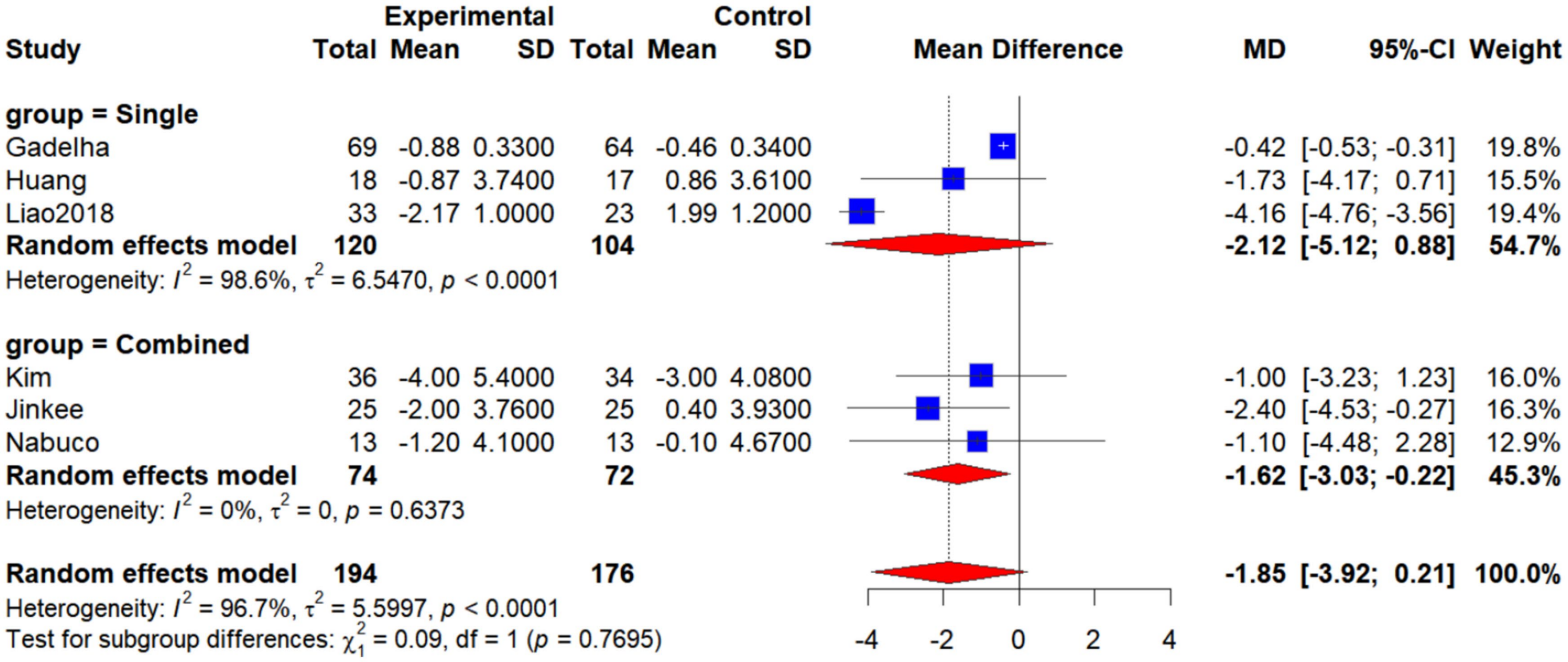
Forest plot of BF% by intervention type (single vs. combined).

Subgroup analysis by intervention type showed that, in the single-intervention group, BF% was lower in the intervention group than in the control group; however, the difference was not statistically significant (WMD = −2.12, 95% CI: −5.12 to 0.88, *I*^2^ = 98.6%, *p* < 0.0001). In the combined-intervention group, BF% was significantly lower in the intervention group compared with the control group (WMD = −1.62, 95% CI: −3.03 to −0.22, *I*^2^ = 0%, *p* = 0.6373). The overall analysis did not demonstrate a statistically significant difference (WMD = −1.85, 95% CI: −3.92 to 0.21, *I*^2^ = 96.7%, p < 0.0001). Tests for subgroup differences indicated no statistically significant differences between the two types of interventions (*χ*^2^ = 0.09, df = 1, *p* = 0.7695; Figure 4). It is noteworthy, however, that although the subgroup difference was not statistically significant (*p* > 0.05), the direction and magnitude of the effect suggest that combined interventions yielded a more consistent and significant reduction in BF% (WMD = −1.62, 95% CI: −3.03 to −0.22, *I*^2^ = 0%), whereas single interventions showed high heterogeneity and nonsignificant effects. This finding implies that combined interventions may be more advantageous than single interventions in reducing BF% among older women with SO (Figure 5).

**Figure 6:**
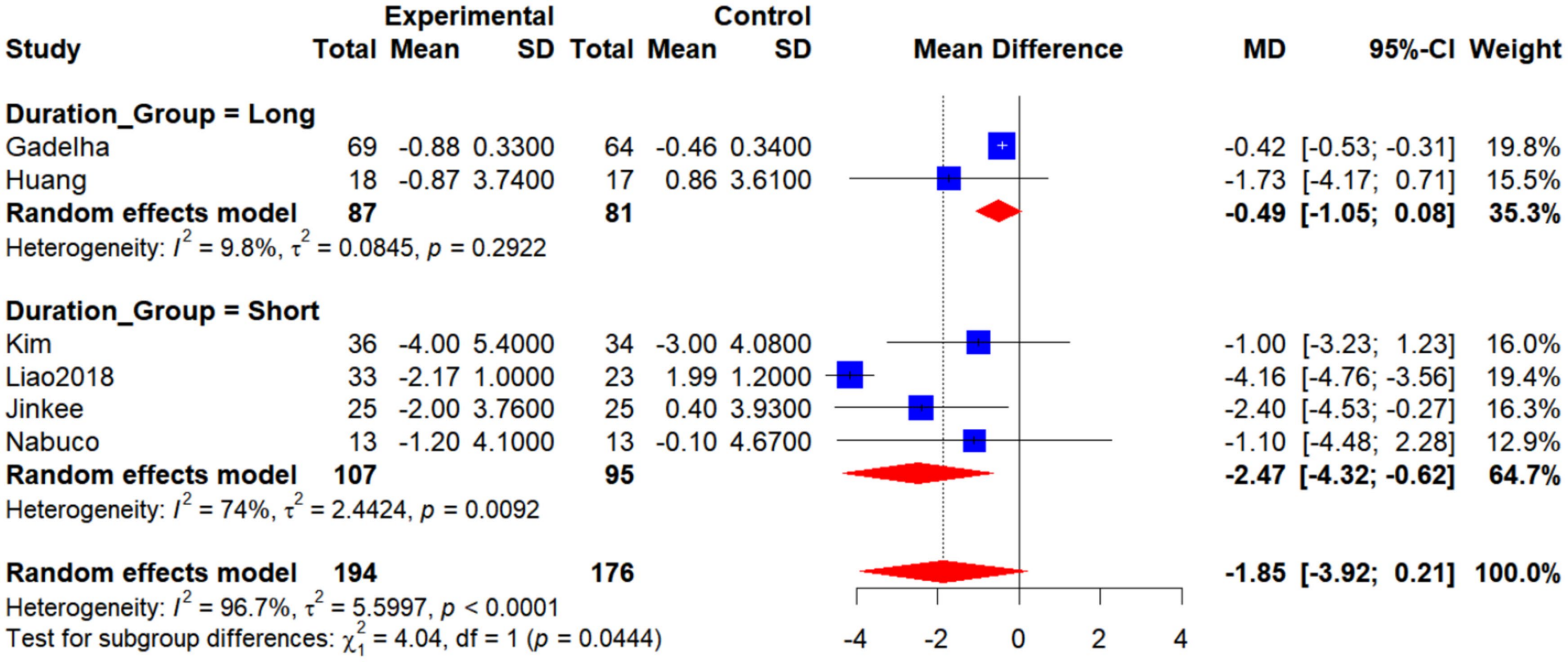
Forest plot of BF% by intervention duration (long vs. short).

Subgroup analyses by intervention duration showed that in long-term interventions (>24 weeks), BF% in the intervention group was lower than in the control group (WMD = −0.49, 95% CI: −4.16 to 3.18, *p* = 0.2922), although the overall effect size was small and heterogeneity was low (*I*^2^ = 9.8%). In contrast, short-term interventions (≤12 weeks) produced a more pronounced reduction in BF% (WMD = −2.47, 95% CI: −4.96 to 0.01, *p* = 0.0092), but with higher heterogeneity (*I*^2^ = 74%). The test for subgroup differences was statistically significant (random-effects model: *χ*^2^ = 5.68, df = 1, *p* = 0.0171), indicating that intervention duration may moderate the effect on BF%, with short-term programs showing greater benefits (Figure 6).

To verify the reliability of these findings, a leave-one-out sensitivity analysis was performed. Sequential exclusion of each individual study yielded pooled WMDs ranging from −2.43 (95% CI: −3.88 to −0.99) to −0.96 (95% CI: −1.87 to −0.05), consistently demonstrating a favorable effect of the intervention. The corresponding heterogeneity statistics (*I*^2^ = 0.2–1.0%) remained largely unchanged, indicating that the overall findings were not disproportionately influenced by any single trial. Excluding Liao et al. (29), led to a slight attenuation of the effect size (WMD = −0.96), yet the statistical significance and direction of the association persisted. Collectively, these results suggest that the evidence for BF% reduction is robust, internally consistent, and insensitive to the omission of individual studies (Supplementary Figure S1).

#### 3.3.2 Analysis of appendicular free fat mass

**Figure 7:**
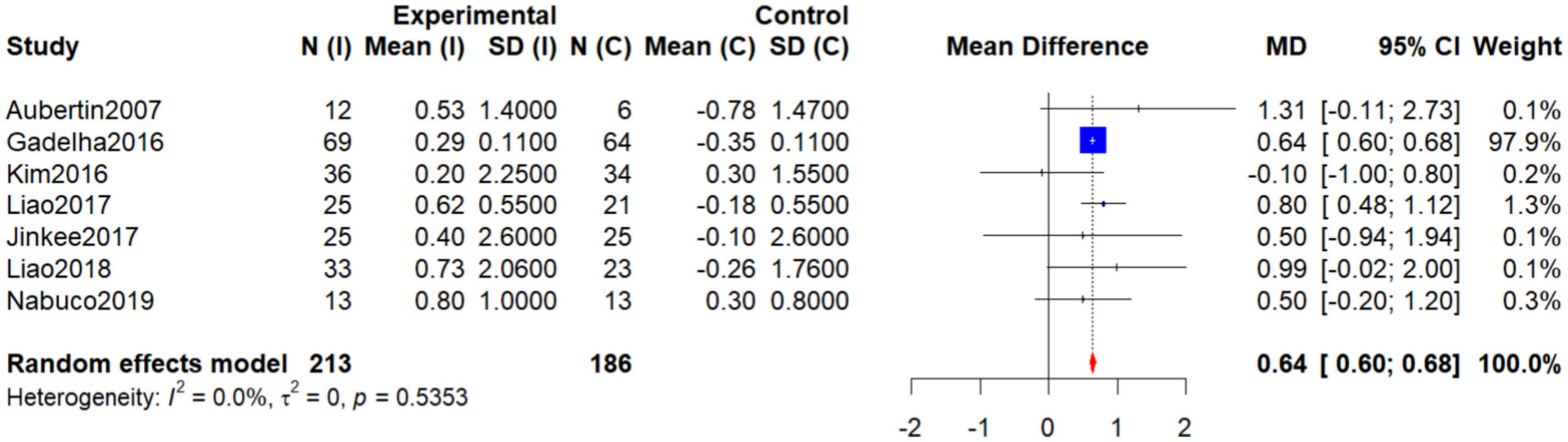
Forest plot of the effect of interventions on AFFM.

Seven studies (19, 22, 27, 29, 30, 33) (Jinkee et al., 2017) evaluated the effects of various non-pharmacological interventions on AFFM in older women with SO. As heterogeneity was negligible (*p* = 0.5353, *I*^2^ = 0%), a random-effects model was applied. The pooled analysis showed that the intervention group had significantly greater AFFM compared with the control group (WMD = 0.64, 95% CI: 0.60–0.68, *p* < 0.001), indicating a robust and consistent positive effect (Figure 7).

Among the included studies, Gadelha et al. (19) contributed the largest sample size, reported stable effects, and carried the greatest weight (97.9%). Liao et al. (27) also reported significant positive results (WMD = 0.80), while Liao et al. (29) found a nearly significant effect (WMD = 0.99), suggesting potential efficacy. Aubertin-Leheudre et al. (22) observed a positive trend (WMD = 1.31) but with wide confidence intervals. By contrast, Kim et al. (33), Jinkee et al. (2017), and Nabuco et al. (30) did not show significant differences, which may be attributed to small sample sizes or large standard deviations.

Overall, the findings consistently support the beneficial effects of non-pharmacological interventions on improving body composition in older women with SO.

Subgroup analysis by intervention type revealed that single-component interventions (e.g., resistance training) produced more pronounced improvements in AFFM (WMD = 0.64, 95% CI: 0.59–0.70), demonstrating consistent benefits across all included studies with no heterogeneity (*I*^2^ = 0%). Among these, the study by Gadelha et al. (19) had the largest sample size, carried the highest weight (97.9%), and showed clear statistical significance. Similarly, Liao et al. (27, 29) reported stable and positive effects (MD = 0.80 and 0.99, respectively). Aubertin-Leheudre et al. (22) also showed marked improvements in the intervention group (MD = 1.31, 95% CI: −0.11 to 2.73), though with wide confidence intervals, suggesting a positive trend.

**Figure 8:**
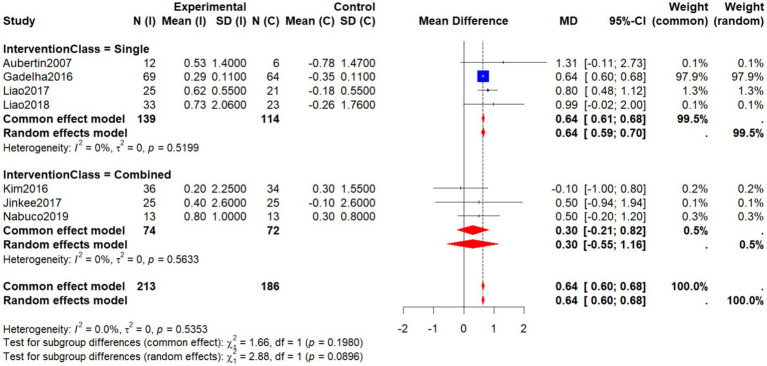
Forest plot of AFFM by intervention type (single vs. combined).

In contrast, combined interventions [e.g., (30, 33)] (Jinkee et al., 2017) yielded effects in the same direction (WMD = 0.30, 95% CI: −0.55 to 1.16), but the results were not statistically significant, although they still suggest potential benefits. The test for subgroup differences was nonsignificant (*p* = 0.0896), further supporting that different nonpharmacological approaches may positively influence AFFM in older women with SO, particularly when considering individualized intervention strategies (Figure 8).

Further subgroup analysis based on nutritional supplementation showed that interventions without additional nutrition demonstrated more consistent and significant benefits. In the four studies without nutritional supplementation (19, 27, 29) (Jinkee et al.2017), the pooled effect size was MD = 0.64 (95% CI: 0.60–0.68), with negligible heterogeneity (*I*^2^ = 0%), indicating a robust positive effect on body composition.

In contrast, studies combining nutritional interventions (22, 33) yielded effects in the same direction but with a lower effect size (MD = 0.43, 95% CI: −1.04 to 1.90) and mild heterogeneity (*I*^2^ = 29.6%). Specifically, Aubertin-Leheudre et al. (22) reported a relatively high mean difference (MD = 1.31) but with wide confidence intervals, while Kim et al. (33) showed a slight negative effect. These findings suggest that combined exercise–nutrition strategies may be influenced by participant characteristics or intervention composition, requiring further validation in future research.

**Figure 9:**
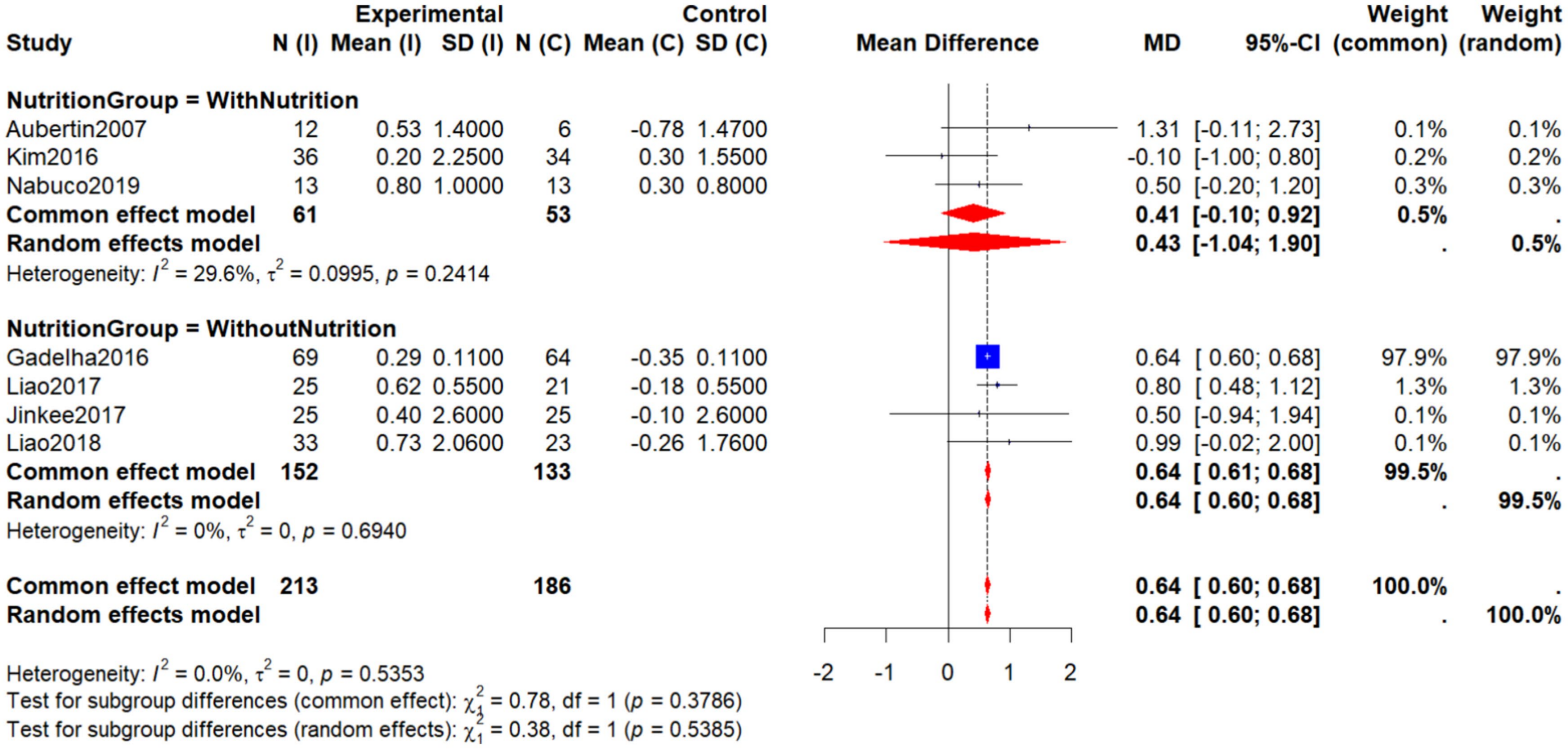
Forest plot of AFFM by nutrition status (with vs. without nutrition).

Although the difference between subgroups was not statistically significant (*χ*^2^ = 0.78, *p* = 0.3786), the evidence indicates that single exercise-based interventions demonstrated clearer and more consistent benefits in this analysis. Overall, regardless of whether nutritional supplementation was included, non-pharmacological interventions showed positive potential in improving AFFM in older women with SO (Figure 9).

**Figure 10:**
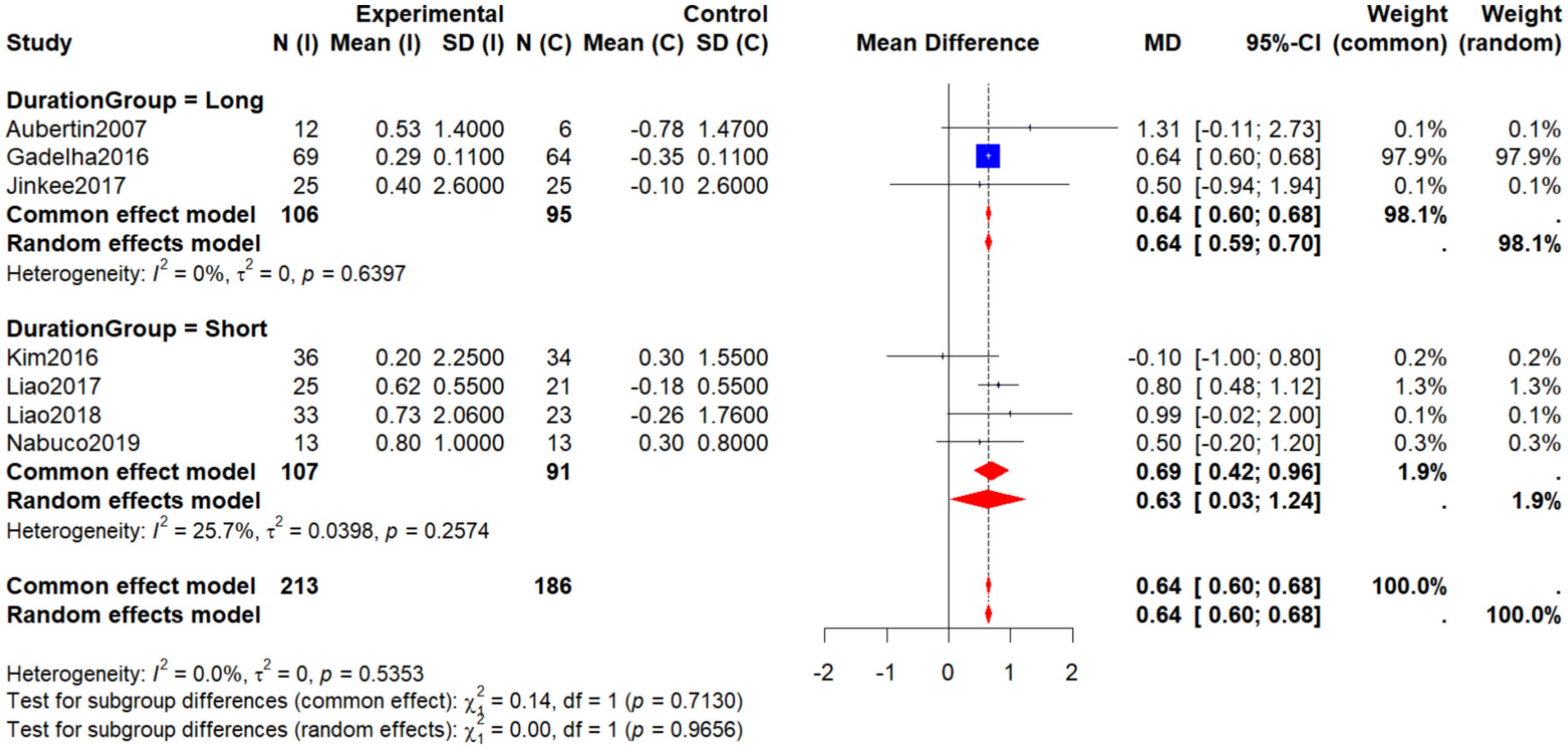
Forest plot of AFFM by intervention duration (long vs. short).

Subgroup analysis by intervention duration divided studies into long-term (≥24 weeks) and short-term (<24 weeks) groups. In the long-term subgroup [three studies: (19, 22); Jinkee et al., 2017], the pooled analysis showed significantly greater AFFM in the intervention group compared with controls (WMD = 0.64, 95% CI: 0.59 to 0.70, *p* < 0.001), with low heterogeneity (*I*^2^ = 0%, *p* = 0.64). In the short-term subgroup [four studies: (27, 29, 30, 33)], the intervention group also demonstrated significantly higher AFFM (WMD = 0.63, 95% CI: 0.03 to 1.24, *p* < 0.05), with moderate heterogeneity (*I*^2^ = 25.7%, *p* = 0.26). The test for subgroup differences was nonsignificant (*χ*^2^ = 0.14, *p* = 0.71), suggesting that intervention duration did not significantly moderate the effects of non-pharmacological interventions on AFFM (Figure 10).

#### 3.3.3 Analysis of handgrip strength

**Figure 11:**
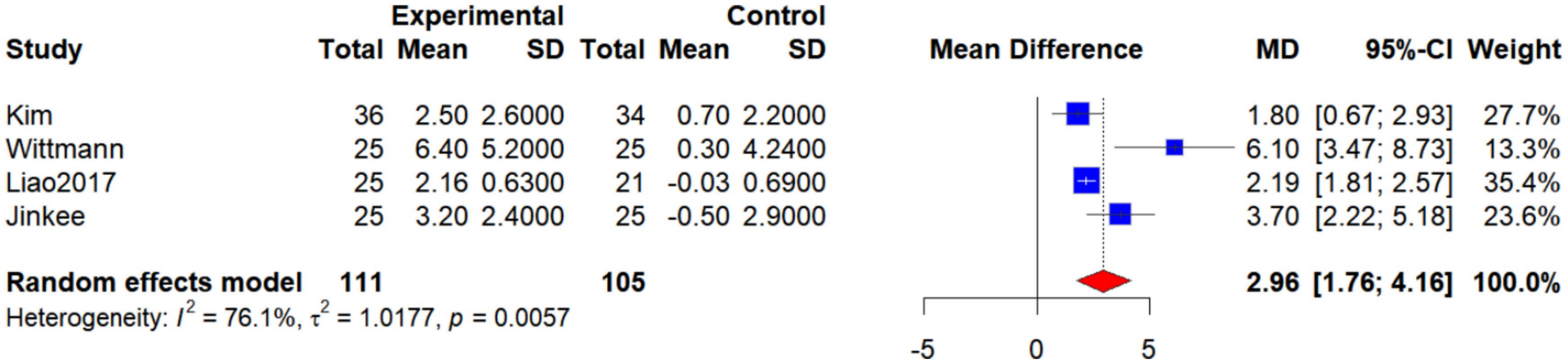
Forest plot of the effect of interventions on HGS.

Four studies (27, 32, 33) (Jinkee et al., 2017) investigated the effects of combined interventions on handgrip strength in older women with SO. Owing to substantial heterogeneity (*p* = 0.0057, *I*^2^ = 76.1%), a random-effects model was used. The pooled analysis demonstrated that handgrip strength was significantly higher in the intervention group compared with the control group (WMD = 2.96, 95% CI: 1.76 to 4.16, *p* < 0.001), indicating that combined interventions can effectively improve muscle strength in this population (Figure 11).

Sensitivity analyses were conducted to assess the influence of individual studies. Excluding Liao et al. (27), and Kim et al. (33) or Jinkee et al. (2017) individually did not alter the significance of the pooled effects (WMDs = 3.68, 3.63, and 3.07, respectively; all 95% CIs not crossing zero; *p* < 0.05), although heterogeneity remained high (*I*^2^ > 83%). However, exclusion of Wittmann et al. (32) reduced the pooled effect size to 2.39 (95% CI: 1.56 to 3.21) and markedly decreased heterogeneity (*I*^2^ = 56.3%, *p* = 0.11), suggesting that this study was the major source of heterogeneity.

#### 3.3.4 Analysis of skeletal muscle index

**Figure 12:**
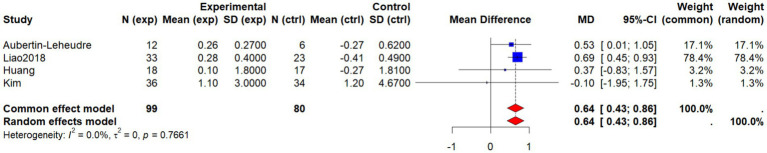
Forest plot of the effect of interventions on SMI.

Four studies (22, 26, 27, 33) investigated the effects of non-pharmacological interventions on skeletal muscle index (SMI) in older women. The meta-analysis showed that SMI was significantly higher in the intervention group compared with controls (WMD = 0.64, 95% CI: 0.43 to 0.86, *p* < 0.001), as presented in (Figure 12). Heterogeneity tests indicated good consistency across studies (*I*^2^ = 0.0%, *p* = 0.7661), suggesting stable and reliable results.

**Figure 13:**
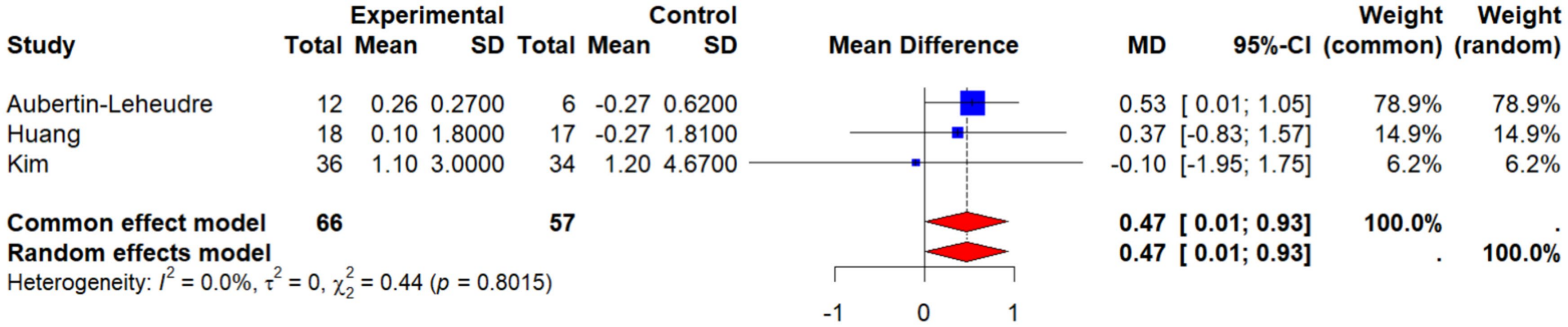
Sensitivity analysis: forest plot of the effect of interventions on SMI.

Among the included studies (27), contributed the highest weight (78.4%) owing to its relatively large sample size and concentrated effect estimates, thereby exerting a decisive influence on the pooled result. To test the robustness of the findings, a sensitivity analysis was conducted by excluding this study. As shown in Figure 13, the remaining three studies yielded a pooled effect of WMD = 0.47 (95% CI: 0.01 to 0.93, *p* = 0.045), which remained statistically significant, with heterogeneity still absent (*I*^2^ = 0%, *p* = 0.8015). This confirmed that the direction and significance of the overall effect were not substantially altered, supporting the robustness of the findings.

#### 3.3.5 Analysis of gait speed

**Figure 14:**
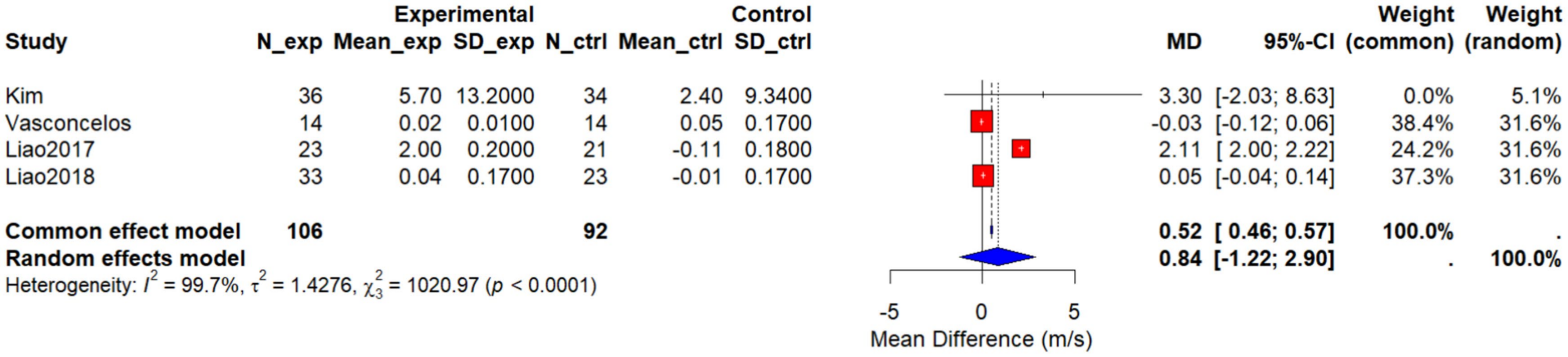
Forest plot of the effect of interventions on GS.

Four studies (25, 27, 29, 33) assessed the effects of non-pharmacological interventions on gait speed (GS) in older women. A pooled analysis using a random-effects model yielded a mean difference (MD) of 0.84 m/s (95% CI: −1.22 to 2.90), which was not statistically significant (*p* = 0.4199). Substantial heterogeneity was observed across studies (*I*^2^ = 99.7%, *τ*^2^ = 1.4276, *p* < 0.0001), indicating marked inconsistency in the findings (Figure 14).

Researchers conducted sensitivity analyses by sequentially excluding individual studies to explore the source of heterogeneity and assess the robustness of the results. As illustrated in Figure 14, exclusion of the Liao et al. (27) study markedly altered the pooled effect, reducing it to 0.01 (95% CI: −0.07 to 0.09), with heterogeneity eliminated (*I*^2^ = 0%, *τ*^2^ ≈ 0). This suggests that the Liao et al. (27) study contributed most substantially to both the overall effect and heterogeneity and was likely the primary source of inconsistency. In contrast, exclusion of the other three studies (25, 29, 33) had minimal impact on the pooled effect, with heterogeneity remaining extremely high (*I*^2^ > 99%).

#### 3.3.6 Analysis of waist circumference

**Figure 15:**
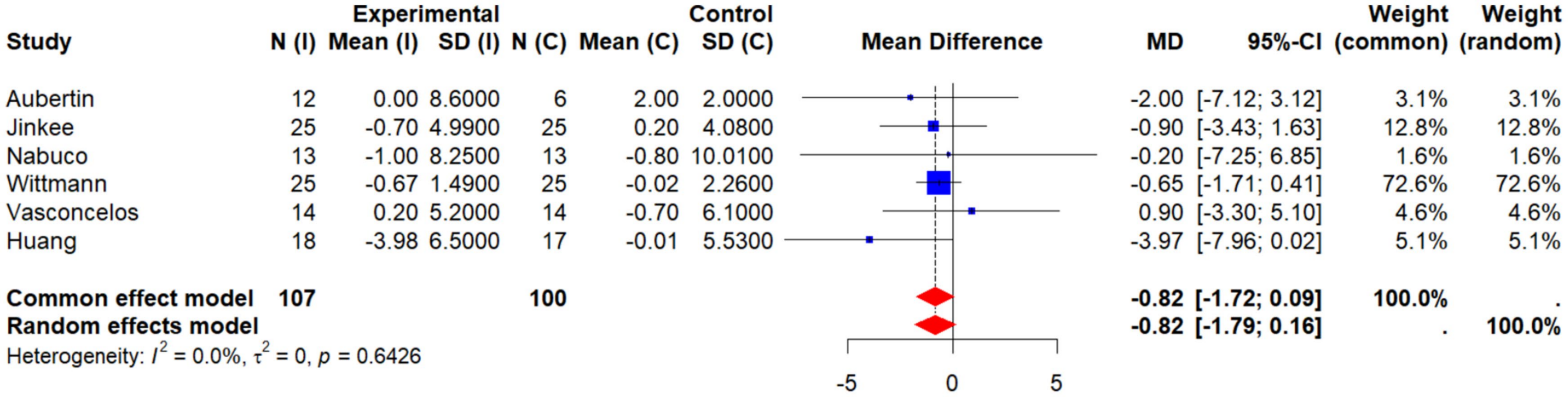
Forest plot of the effect of interventions on WC.

This meta-analysis included six studies to evaluate the effects of non-pharmacological interventions on waist circumference (WC) in older adults. A pooled analysis using a random-effects model showed a mean difference (MD) of −0.82 (95% CI: −1.79 to 0.16) between the intervention and control groups, which was not statistically significant (*p* > 0.05). Between-study heterogeneity was low and not statistically significant (*I*^2^ = 0.0%, *p* = 0.6426), suggesting that the direction and magnitude of effects were relatively consistent across studies (Figure 15).

**Figure 16:**
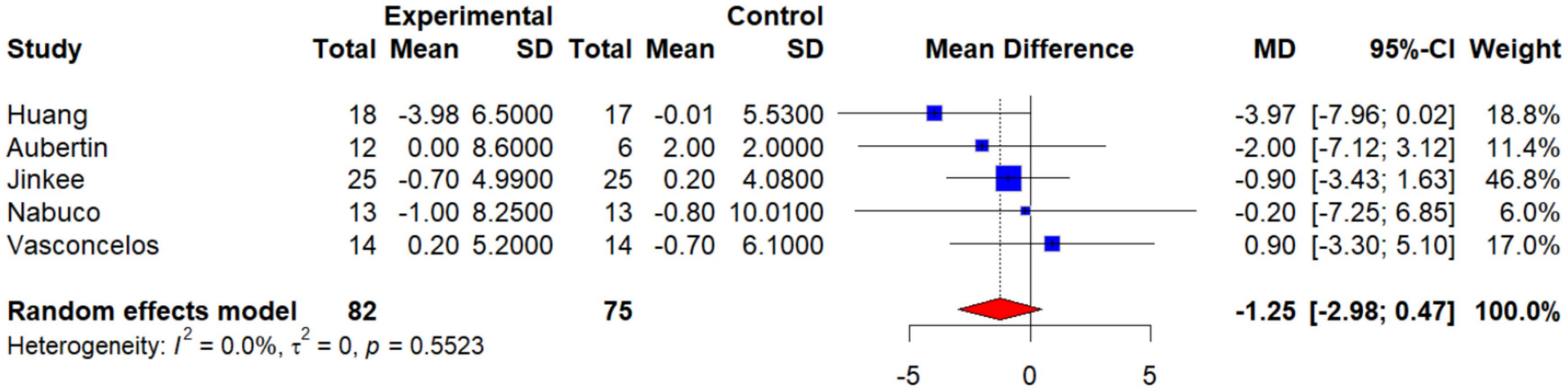
Sensitivity analysis: forest plot of the effect of interventions on WC.

To further examine how individual studies might influence the overall effect, researchers performed a sensitivity analysis by excluding the Wittmann study, which contributed the largest weight (72.6%). After exclusion, the pooled effect was MD = −1.25 (95% CI: −2.98 to 0.47), which still did not reach statistical significance. Heterogeneity remained unchanged (*I*^2^ = 0.0%, *p* = 0.5523), indicating that the results were robust and not dominated by a single study (Figure 16).

#### 3.3.7 Analysis of body mass index

A total of four randomized controlled trials (RCTs) involving 135 participants in the intervention groups and 121 in the control groups were included to assess the effects of exercise interventions on body mass index (BMI). Meta-analysis revealed no statistically significant difference in BMI changes between the two groups (MD = −0.93, 95% CI: −3.12 to 1.26, *p* > 0.05), suggesting that the overall impact of exercise interventions on BMI was limited.

Subgroup analyses demonstrated that neither single interventions (MD = −0.12, 95% CI: −0.61 to 0.38; *I*^2^ = 0%) nor combined interventions (MD = −1.84, 95% CI: −5.10 to 1.41; *I*^2^ = 86.2%) exerted a significant influence on BMI. Furthermore, no significant differences were observed between subgroups (*χ*^2^ = 1.06, df = 1, *p* = 0.3041), indicating that intervention type was not a decisive factor in BMI outcomes.

**Figure 17:**
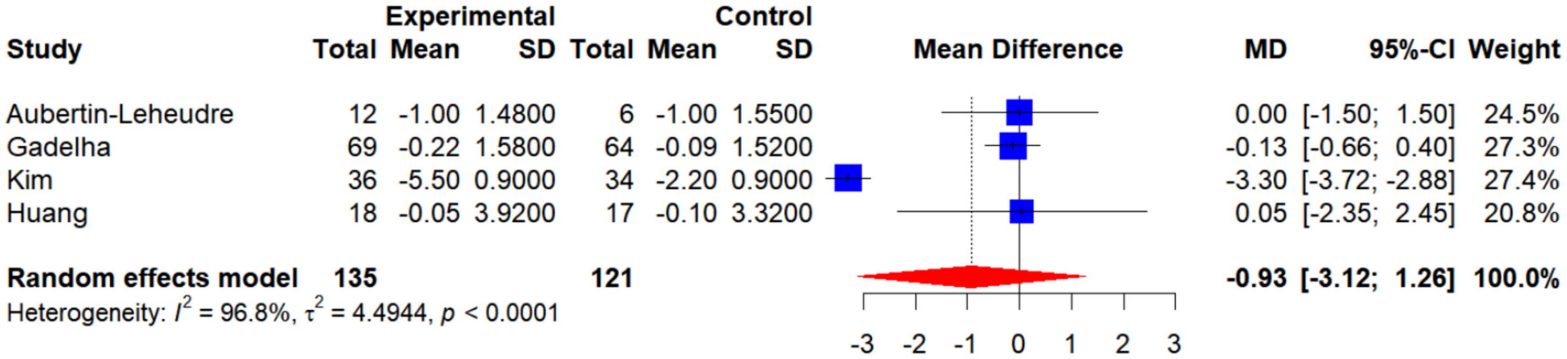
Forest plot of the effect of interventions on BMI.

**Figure 18:**
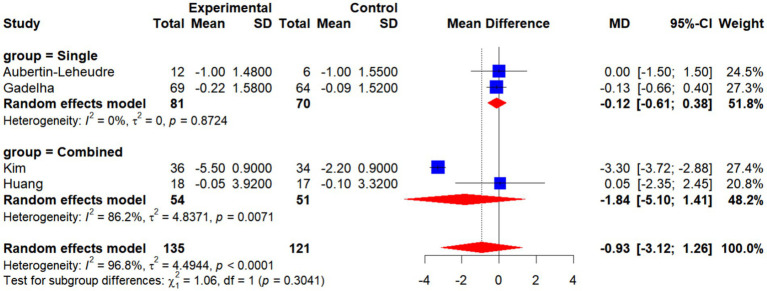
Forest plot of BMI by intervention type (single vs. combined).

Taken together, current evidence does not support a significant effect of exercise interventions on BMI in older women with SO. These findings highlight the need for further validation through large-scale, high-quality trials (Figures 17, 18).

#### 3.3.8 Summary of evidence on aquatic exercise

In addition to conventional land-based training, aquatic exercise has been explored as a non-pharmacological intervention for older women with SO. The buoyancy of water reduces joint stress and lowers the risk of injury, while the inherent resistance of water enhances muscle activation and energy expenditure. Evidence from existing trials suggests that aquatic exercise can improve body composition and physical function (31); however, these effects have not consistently reached statistical significance, likely due to small sample sizes and short intervention durations. Despite these limitations, aquatic exercise appears to be a safe and feasible alternative for individuals with mobility impairments or joint discomfort, underscoring the need to tailor intervention strategies to the specific needs of older women with SO.

## 4 Discussion

### 4.1 Overall findings

This is the first meta-analysis of randomized controlled trials evaluating the effects of non-pharmacological interventions on SO in older women. A comprehensive search across multiple databases was conducted with rigorous inclusion and exclusion criteria. Overall, the findings consistently indicated that non-pharmacological interventions significantly improved BF%, AFFM, SMI, and handgrip strength when compared with controls. In contrast, no significant changes were observed in BMI, WC, or GS. Although variations in training type, duration, intensity, and sample size influenced the observed outcomes, the generally high methodological quality of the included studies provides sufficient evidence to support the application of non-pharmacological interventions in older women with SO. Beyond differences in intervention protocols, variations in the diagnostic definitions of sarcopenic obesity (SO) also emerged as a major source of heterogeneity (Supplementary Table S1). The majority of studies [e.g., (19, 22, 25–27, 30)] employed self-established or researcher-defined criteria rather than adopting internationally harmonized guidelines. Only a small number (27, 32) followed the EWGSOP 2010 (EWGSOP1) recommendations, and one study (33) utilized the AWGS 2014 operational definition tailored to Asian populations. Specifically, studies based on EWGSOP1 emphasized quantitative loss of skeletal muscle mass, whereas those following AWGS 2014 incorporated muscle strength and gait performance, thereby enrolling participants with more evident functional limitations. Conversely, studies employing researcher-defined cut-offs tended to rely solely on BF%, BMI, or appendicular lean mass, without evaluating neuromuscular function. This diagnostic heterogeneity likely contributed to inconsistencies across outcomes. For instance, several trials reported significant gains in handgrip strength, whereas changes in BF% or BMI remained negligible, which may have accounted for a substantial portion of the heterogeneity observed across pooled analyses. To enhance the comparability and interpretability of future studies, adopting standardized diagnostic frameworks—such as EWGSOP 2019 or AWGS 2019—that integrate structural, functional, and metabolic components of sarcopenia will be essential for establishing a unified basis for cross-study synthesis.

Building upon the above findings regarding diagnostic variability and overall intervention effects, this meta-analysis further examines both overall and subgroup outcomes across multiple indicators. This approach enables a more integrated understanding of how intervention type, duration, and diagnostic framework influence changes in body composition and physical function among older women with SO.

#### 4.1.1 Fat-related outcomes

Non-pharmacological interventions significantly reduced BF%, with combined interventions producing greater improvements than single-modality interventions, suggesting the superiority of multimodal approaches. Such combined strategies typically included resistance training plus aerobic exercise (enhancing basal metabolic rate and energy expenditure) and resistance training plus nutritional supplementation (e.g., protein or isoflavones). The former improves fat oxidation via increased basal metabolic rate (BMR) and insulin sensitivity, while the latter promotes protein synthesis and higher metabolic activity to optimize body composition (34–36). Of note, Aubertin et al. (22) applied isoflavone supplementation, which differs from conventional protein or amino acid supplementation but aligns with the study’s focus on older women with SO. Isoflavones, acting as phytoestrogens, may improve fat distribution and metabolic status, thereby indirectly lowering BF%. Thus, although this intervention differs mechanistically from standard protein supplementation, it still qualifies as a non-pharmacological approach and offers valuable insights into the role of diverse nutritional strategies in SO management.

Subgroup analysis revealed that short-term interventions produced more pronounced reductions in BF%, possibly due to rapid early metabolic adaptations and energy deficits, whereas long-term effects appeared more modest Beyond physiological mechanisms, this pattern may also reflect behavioral and psychological dynamics—participants in early intervention phases often demonstrate higher motivation, stronger adherence, and closer supervision, while long-term programs may struggle to sustain engagement and regular participation (37, 38). Consequently, the attenuated effects observed over time may stem less from reduced biological efficacy and more from declines in motivation and adherence fatigue, both of which are well-documented challenges in lifestyle and physical activity interventions (39, 40). Higher heterogeneity in short-term interventions may be attributable to differences in intervention type, intensity, baseline adiposity, and adherence. In contrast, combined interventions with more standardized protocols yielded lower heterogeneity, reflecting more stable outcomes. Clinically, the present study underscores the value of short-term multimodal interventions to improve BF% in older women with SO, especially when exercise is paired with tailored nutritional supplementation to enhance adherence and effectiveness. To further validate the robustness of these results, a leave-one-out sensitivity analysis was performed for BF%. Sequential exclusion of each study yielded consistently negative pooled effects, indicating that the significant reduction in BF% was not driven by any single trial. Although exclusion of (29) slightly attenuated the effect size, the overall direction and statistical significance remained unchanged, suggesting that the observed improvement in BF% is robust and internally consistent despite between-study heterogeneity.

#### 4.1.2 Other body composition measures

No significant improvements were observed in WC, possibly due to insufficient intervention duration or limited sample size. Similarly, BMI remained unchanged, reflecting its insensitivity to body composit4.

ion shifts. When fat loss and muscle gain occur simultaneously, BMI changes may cancel each other out (41–44). Therefore, WC and BMI should be interpreted alongside BF%, SMI, and AFFM to provide a more comprehensive evaluation of intervention efficacy.

#### 4.1.3 Muscle mass outcomes

Both AFFM and SMI improved significantly, with stable results and low heterogeneity. AFFM appeared to be more sensitive to intervention modality. Resistance training alone consistently improved AFFM, whereas combined interventions showed comparable but slightly smaller effect sizes and greater heterogeneity. Subgroup analysis revealed no significant differences between combined interventions with and without nutritional supplementation, consistent with previous evidence (45, 46). Although combined interventions incorporating nutritional supplementation were more effective in reducing BF% than exercise alone, they did not produce a significant increase in AFFM. This discrepancy likely reflects the distinct physiological mechanisms that govern fat reduction and muscle hypertrophy. Protein-or isoflavone-based supplements may promote fat oxidation through hormonal modulation and enhanced thermogenesis, facilitating fat loss without necessarily inducing substantial muscle accretion (47, 48). In contrast, meaningful gains in lean mass require strong anabolic stimuli derived from mechanical loading, adequate training intensity, and sufficient intervention duration (49). Variations in supplement type, dosage, and participant adherence across studies may also contribute to inconsistent outcomes. Overall, these findings suggest that supplementation may enhance fat metabolism and improve body composition, but its anabolic efficacy largely depends on concurrent high-intensity resistance training and sufficient protein intake. This interpretation reconciles the observed discrepancy between BF% reduction and AFFM change, emphasizing the need for integrative designs that coordinate nutritional and exercise components to optimize outcomes in older women with sarcopenic obesity. Furthermore, the findings suggest that standardized resistance training provides sufficient anabolic stimulation, with additional nutritional supplementation conferring no short-term statistical advantage. Intervention duration analysis showed that both short-term (<24 weeks) and long-term (≥24 weeks) protocols significantly increased AFFM, with no meaningful differences between them. This indicates that short-term interventions are effective for rapid gains, supporting adherence, while long-term programs remain valuable for sustaining muscle mass and preventing relapse.

SMI, a height-adjusted relative measure of muscle mass, also improved significantly post-intervention. Unlike AFFM, SMI improvements may be partly driven by concurrent fat reduction, corroborating trends observed in BF%. Its low heterogeneity suggests high consistency across studies and populations, underscoring its robustness. Higher SMI has been associated with increased BMR and favorable fluid balance ratios (lower Extracellular Water / Intracellular Water and Extracellular Water / Total Body Water) (24), reflecting improvements in muscle mass, function, and metabolic health. Clinically, greater SMI is linked to enhanced functional capacity, reduced fall risk, and better quality of life (3). Thus, resistance training should remain central in SO management, with future research examining the sustainability of SMI improvements under different training regimens and cycles.

#### 4.1.4 Functional outcomes

Handgrip strength improved significantly, indicating the efficacy of combined interventions in enhancing muscle strength. Most included studies employed multimodal protocols, such as resistance plus aerobic training or nutritional supplementation, with some incorporating electrical stimulation. Sensitivity analysis identified Wittmann et al. as a key source of heterogeneity, as their use of electrical stimulation differed from conventional exercise. Electrical stimulation can directly induce muscle contraction, improve local circulation, and activate fast-twitch fibers, resulting in short-term gains in grip strength (50). Consistent with the main results, the sensitivity analysis showed that excluding Wittmann et al. (32), which used whole-body EMS, substantially reduced heterogeneity while preserving the overall significance and direction of the pooled effect. This finding confirms that the positive effect of non-pharmacological interventions on handgrip strength remains stable and reliable even after accounting for methodological variation. These results suggest that electrical stimulation may be a promising adjunct, particularly for SO women with joint pain, cardiopulmonary limitations, or poor adherence to conventional exercise. A systematic review has also reported that whole-body EMS significantly enhances muscle mass and function (grip strength, gait speed) in older adults (51), particularly among those unable to tolerate high-intensity exercise. However, current evidence is limited, and optimal parameters (frequency, intensity, and duration) remain unclear, warranting further high-quality RCTs.

For GS, no overall significant improvements were observed, accompanied by high heterogeneity. Sensitivity analysis identified the study by Liao et al. (27) as the main source of heterogeneity, as its elastic-band resistance training produced substantially larger gains than those reported in other studies. Elastic-band training provides progressive resistance with low joint loading, balancing safety and feasibility while enhancing lower-limb strength, joint flexibility, and neuromuscular coordination. These factors may jointly improve gait control and mobility (33, 52). While evidence remains preliminary, elastic-band training shows promise for improving GS in SO women, particularly those with joint impairments or poor tolerance for high-load training, and should be further investigated in future trials. Although muscle mass and handgrip strength improved significantly, these gains may not translate rapidly into faster gait speed (GS) because walking performance is influenced by more than just strength or mass. For example, age-related changes in neuromuscular control, such as wider activation of lumbar and sacral motor pools, altered muscle activation timing, and reduced muscle–tendon stiffness, have been shown to impair gait mechanics and propulsion (53). In the present meta-analysis, most included interventions appear to have emphasised general or upper-body and whole-body resistance training rather than specific lower-limb push-off or power-based exercises (e.g., fast ankle/knee movements, gait-specific drills). Such omission may limit improvements in distal propulsion, which is critical for increasing gait speed in older adults.

Thus, the combination of neuromuscular/biomechanical constraints and an intervention focus that may have under-targeted lower-limb power helps explain why no significant improvement in GS was observed despite improvements in muscle mass and strength.

#### 4.1.5 Additional evidence

This review also included a quasi-experimental study (31) using aquatic exercise. Despite its non-RCT design, inclusion was justified by its SO female sample and alignment of outcomes with this analysis. Aquatic exercise reduces joint stress via buoyancy while combining resistance and aerobic benefits, making it particularly suitable for older women with SO who have joint limitations or cannot tolerate high-load land-based training (54–57). These findings support aquatic exercise as a safe and feasible adjunct in clinical and community-based interventions, though further validation from high-quality RCTs is needed.

Behavioral and Educational Perspectives for Sustainable Management.

Beyond the physiological mechanisms discussed above, it is equally important to recognize the behavioral and educational dimensions that determine whether intervention benefits can be maintained over time. Although this meta-analysis demonstrates clear physiological improvements in body composition and muscle strength among older women with sarcopenic obesity, such effects are unlikely to persist without continuous behavioral engagement and health-related learning support. Effective management of sarcopenic obesity should therefore be understood not as a short-term exercise protocol but as an ongoing process of guided lifestyle learning and self-regulation.

In practice, the short-term physiological gains—such as fat reduction or muscle growth—represent only the initial adaptation stage. Without reinforcement through education and motivation, participants often revert to sedentary habits, diminishing the achieved benefits. Hence, clinical and community-based programs should integrate structured exercise and nutritional interventions with behavioral components that strengthen intrinsic motivation, self-efficacy, and long-term adherence. For example, participants can be encouraged to set realistic goals, track progress, and apply problem-solving strategies to maintain activity in daily life. Moving from a “training-for-outcome” model toward an “education-for-sustainability” approach is essential for ensuring durable health benefits. This integrated framework combines physiological, behavioral, and cognitive dimensions, fostering not only biological adaptation but also sustainable behavioral change. Future studies should develop hybrid intervention models that embed physical training within continuous health education, enabling older women to sustain self-directed, health-promoting behaviors beyond the intervention period.

## 5 Conclusion

This meta-analysis demonstrates that non-pharmacological interventions, particularly resistance-based or combined exercise programs, are effective and safe strategies for improving body composition (BF%, AFFM, SMI) and selected aspects of physical function, notably handgrip strength and muscle mass, in older women with SO. Resistance training remains the cornerstone of these interventions, while the integration of aerobic exercise further promotes fat reduction and muscle hypertrophy. However, improvements in BMI, waist circumference, and gait speed were less consistent, indicating that responses may vary by outcome type and intervention duration. Despite the predominance of female participants in existing studies, research specifically targeting older women with SO is still limited. Furthermore, most interventions were short-term and focused on traditional exercise, with insufficient evaluation of emerging modalities such as electrical stimulation, isoflavone supplementation, and aquatic exercise.

## 6 Recommendations

Future research should focus on determining the most appropriate timing and duration of non-pharmacological interventions for older women with SO. Well-designed randomized controlled trials are required to compare short-term and long-term intervention effects and to verify the effectiveness of a “short-term intensification followed by long-term maintenance” model. Short-term programs (fewer than 24 weeks) may emphasize combined approaches—resistance training, aerobic exercise, and personalized nutrition—to achieve rapid improvements in muscle gain and fat reduction. In contrast, long-term programs (24 weeks or longer) should highlight consistent resistance training supported by nutritional management and adjunctive strategies such as electrical stimulation or aquatic exercise to sustain benefits. Developing an evidence-based framework for intervention scheduling will help establish practical, individualized, and sustainable treatment strategies for this population. From a clinical and public health standpoint, integrating structured resistance-based interventions into community health programs may serve as an effective strategy to reduce frailty, improve metabolic health, and maintain functional independence in older women with SO. Importantly, future interventions should transition from isolated exercise programs to continuous, education-oriented processes that foster self-regulation, intrinsic motivation, and health literacy. Integrating behavioral education into exercise and nutrition frameworks will not only strengthen adherence but also enhance the long-term sustainability of intervention outcomes at both the individual and population levels.

## Data Availability

The original contributions presented in the study are included in the article/Supplementary material, further inquiries can be directed to the corresponding author/s.
