# Supplementary figures and images for: Effects of non-pharmacological interventions on body composition and physical function in older women with sarcopenic obesity: a meta-analysis
Source: Front Public Health. 2025 Dec 12;13:1718720. doi: 10.3389/fpubh.2025.1718720 (PMC12741268; doi:10.3389/fpubh.2025.1718720)

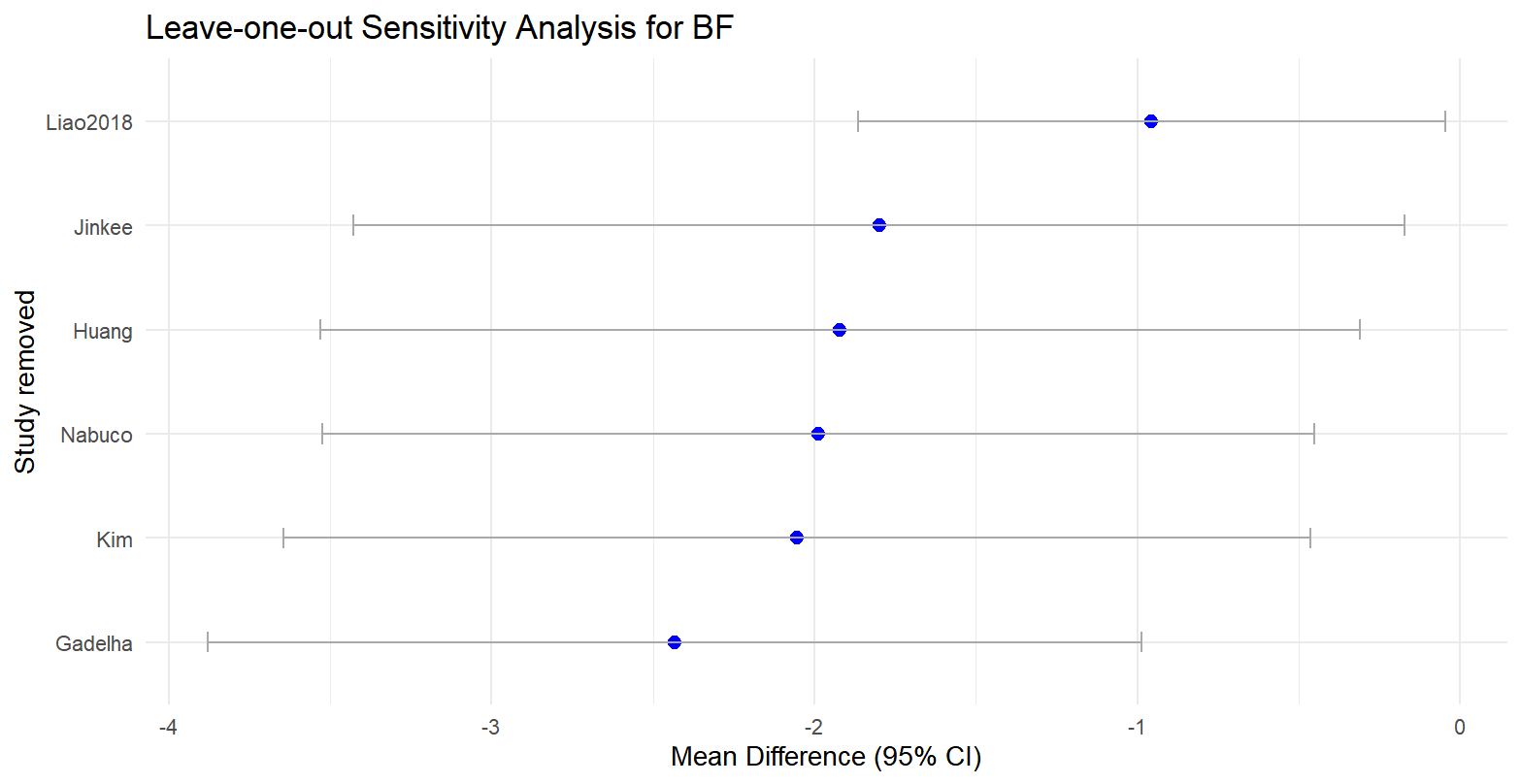

Supplement: Supplementary file 1 [file Image_1.png]
