# Supplementary material for: Effects of non-pharmacological interventions on body composition and physical function in older women with sarcopenic obesity: a meta-analysis
Source: Front Public Health. 2025 Dec 12;13:1718720. doi: 10.3389/fpubh.2025.1718720 (PMC12741268; doi:10.3389/fpubh.2025.1718720)
Supplement: Supplementary file 2 [file Supplementary_file_1.doc]

**Table 1. Search strategy for database.**

| Pubmed（2025-4-25） | | Total |
| --- | --- | --- |
| #1 | **(((Obesity[MeSH Terms]) OR (Obesity)) AND ((Sarcopenia[MeSH Terms]) OR (sarcopenia))) OR ((((sarcobesity) OR (obese sarcopen*)) OR (sarcopenia obes*)) OR ("Sarcopenia obesity"))** | 3705 |
| #2 | **(((aged[MeSH Terms]) OR (elderly)) OR (senior)) OR (old adult)** | [6,729,887](https://pubmed.ncbi.nlm.nih.gov/?term=(((aged%5BMeSH+Terms%5D)+OR+(elderly))+OR+(senior))+OR+(old+adult)&sort=&size=50) |
| #3 | **(Female[MeSH Terms]) OR (Females)** | [10,416,693](https://pubmed.ncbi.nlm.nih.gov/?term=(Female%5BMeSH+Terms%5D)+OR+(Females)&sort=&size=50) |
| #4 | (("Exercise"[Mesh]) OR ((((((Physical Exercise) OR (Aerobic Exercise)) OR (Isometric Exercise)) OR (Acute Exercise)) OR (Exercise Training)) OR (Physical Activity))) OR (((((((((((Diet, Food, and Nutrition[MeSH Terms]) OR (Nutrition Therapy[MeSH Terms])) OR (exercise)) OR (diet)) OR (nutr*)) OR (energy restriction)) OR (Electric Stimulation Therapy[MeSH Terms])) OR (Electrotherapy)) OR (Therap* Electrical Stimulation)) OR (Electrical Stimulation, Therap*)) OR (Interferential Current Electrotherapy)) | 3058590 |
| #5 | #1 AND #2 AND #3 AND #4 | 921 |

| Corchrane（2025-4-25） | | Total |
| --- | --- | --- |
| #1 | MeSH descriptor: [Sarcopenia] explode all trees | 992 |
| #2 | MeSH descriptor: [Obesity] explode all trees | 21557 |
| #3 | ((sarcobesity) OR (obese sarcopen*) OR (sarcopenia obes*) OR ("Sarcopenia obesity")):ti,ab,kw | 369 |
| #4 | #1 and #2 or #3 | 370 |
| #5 | MeSH descriptor: [Aged] in all MeSH products | 276051 |
| #6 | (elderly or senior or old adult):ti,ab,kw | 94110 |
| #7 | #5 or #6 | 345723 |
| #8 | MeSH descriptor: [Female] in all MeSH products | 606695 |
| #9 | (females):ti,ab,kw | 996478 |
| #10 | #8 or #9 | 996478 |
| #11 | #4 and #7 and #10 | 101 |
| #12 | MeSH descriptor: [Exercise] explode all trees | 39411 |
| #13 | MeSH descriptor: [Diet, Food, and Nutrition] explode all trees | 76266 |
| #14 | MeSH descriptor: [Nutrition Therapy] explode all trees | 12747 |
| #15 | MeSH descriptor: [Electric Stimulation Therapy] explode all trees | 11243 |
| #16 | (Physical Exercise OR Aerobic Exercise OR Isometric Exercise OR Acute Exercise OR Exercise Training OR Physical Activity):ti,ab,kw | 149502 |
| #17 | (nutr* or diet or energy restriction):ti,ab,kw | 127702 |
| #18 | (Electrotherapy OR Therap* Electrical Stimulation OR Electrical Stimulation, Therap* OR Interferential Current Electrotherapy):ti,ab,kw | 10544 |
| #19 | #13 or #14 or #15 or #16 or #17 or #18 | 307008 |
| #20 | #19 and #11 | 80 |

| wos（2025-4-25） | | Total |
| --- | --- | --- |
| #1 | TS=(Sarcopenia and Obesity) | 6307 |
| #2 | TS=(sarcobesity OR obese sarcopen* OR sarcopenia obes* OR "Sarcopenia obesity") | [6741](https://pubmed.ncbi.nlm.nih.gov/?term=(((aged%5BMeSH+Terms%5D)+OR+(elderly))+OR+(senior))+OR+(old+adult)&sort=&size=50) |
| #3 | TS=(elderly or senior or old adult or aged) | 11305452 |
| #4 | TS=(female or females) | 12532932 |
| #5 | #4 AND #3 AND #2 AND #1 | 3312 |
| #6 | TS=(exercise OR Physical Exercise OR Aerobic Exercise OR Isometric Exercise OR Acute Exercise OR Exercise Training OR Physical Activity OR nutr* OR diet OR energy restriction OR Nutrition Therapy OR Diet, Food, and Nutrition OR Electrotherapy OR Therap* Electrical Stimulation OR Electrical Stimulation, Therap* OR Interferential Current Electrotherapy OR Electric Stimulation Therapy ) | 4753864 |
| #7 | #5 AND #6 | 2288 |

| embase（2025-4-25） | | Total |
| --- | --- | --- |
| #1 | **'sarcopenia'**/exp OR **'sarcopenia'** | 37564 |
| #2 | **'obesity'** | [82864](https://pubmed.ncbi.nlm.nih.gov/?term=(((aged%5BMeSH+Terms%5D)+OR+(elderly))+OR+(senior))+OR+(old+adult)&sort=&size=50) |
| #3 | #1 AND #2 | 6395 |
| #4 | **'sarcobesity'** OR **'obese sarcopen*'** OR **'sarcopenia obes*'** OR **'sarcopenia obesity'** | 328 |
| #5 | #3 OR #4 | 6420 |
| #6 | **'aged'**/exp OR **'aged' OR 'elderly'** OR **'senior' OR 'old adult'** | 6603336 |
| #7 | **'female'**/exp OR **'female'** | 13443674 |
| #8 | #5 AND #7 AND #8 | 87140 |
| #9 | **'exercise'**/exp OR **'exercise'** | 776678 |
| #10 | **'nutrition'**/exp OR **'nutrition'** | 3440188 |
| #11 | **'diet therapy'/exp OR 'diet therapy'** | 468548 |
| #12 | **'electrotherapy'/exp OR 'electrotherapy'** | 360743 |
| #13 | **'physical exercise':ti,ab,kw OR 'aerobic exercise':ti,ab,kw OR 'isometric exercise':ti,ab,kw OR 'acute exercise':ti,ab,kw OR 'exercise training':ti,ab,kw OR 'physical activity':ti,ab,kw**  **OR 'diet, food, and nutrition':ti,ab,kw OR 'nutrition therapy':ti,ab,kw OR 'nutr*':ti,ab,kw OR 'diet':ti,ab,kw OR 'energy restriction':ti,ab,kw OR 'electrotherapy':ti,ab,kw OR 'therap* electrical stimulation':ti,ab,kw OR 'electrical stimulation, therap*':ti,ab,kw OR 'interferential current electrotherapy':ti,ab,kw** | 1475066 |
| #14 | #9 OR #10 OR #11 OR #12 OR #13 | 4788619 |
| #15 | #8 AND #14 | 1563 |
